# Dynamic changes in metabolic and lipidomic profiles of tea plants during drought stress and re-watering

**DOI:** 10.3389/fpls.2022.978531

**Published:** 2022-09-02

**Authors:** Jiazhi Shen, Shuangshuang Wang, Litao Sun, Yu Wang, Kai Fan, Chen Li, Hui Wang, Caihong Bi, Fen Zhang, Zhaotang Ding

**Affiliations:** ^1^Tea Research Institute, Shandong Academy of Agricultural Sciences, Jinan, China; ^2^Tea Research Institute, Qingdao Agricultural University, Qingdao, China; ^3^Tea Research Institute, Rizhao Academy of Agricultural Sciences, Rizhao, China; ^4^Linyi Agricultural Technology Extension Center, Linyi, China; ^5^Agriculture and Rural Affairs Bureau of Wulian County, Rizhao, China

**Keywords:** *Camellia sinensis* L., drought stress, lipidomic, metabolomic, re-water

## Abstract

Tea (*Camellia sinensis* L.), as an evergreen plant, needs a humid environment. Water deficit would diminish tea yield and quality. We analyzed the dynamic changes in the metabolite and lipid profiling of tea leaves under various drought conditions and re-watering to determine the metabolic changes in tea leaves responding to drought challenges. In all, 119 metabolites showed substantial alterations in drought-stressed tea plants, including sugars and sugar alcohols, amino acids, and tricarboxylic acid cycle intermediates and lipids. We detected 29 lipids and they were classified into phosphatidylglycerol (PG), phosphatidic acid (PA), sulfoquinovosyl-diacylglycerol (SQDG), phosphatidylcholine (PC), lyso-phosphatidylcholine (LysoPC), and phosphatidylinositol (PI). The levels of sugar, sugar alcohol, and sugar precursors may change as a response to drought stress. Compared with these metabolites, the membrane lipids showed more dynamic changes in tea under drought stresses. Furthermore, metabolic recovery was only partial, with the majority of the examined metabolites exhibiting significantly different levels between samples from re-watered and well-watered tea plants. The findings also showed that comprehensive metabolomic and lipidomic approaches were efficient in elucidating the impacts of drought stress on tea plant metabolism. Our findings are valuable for understanding the mechanisms behind drought tolerance in tea plants from the metabolism perspective and utilizing the compounds to improve the drought tolerance of tea plants.

## Introduction

Tea (*Camellia sinensis* L.), containing specialized metabolites contributing to rich flavors and healthy function, is an important economic crop in tropical and subtropical regions, and is typically cultivated in a rain-fed agricultural system. However, because of its adaptability to the original favorable environment, the tea plant is adversely affected by a variety of abiotic stressors. Tea yields are greatly influenced by weather, especially by drought, which causes irreparable losses (Chaves et al., 2003; Cheruiyot et al., 2007; Xu et al., 2010b; Upadhyaya et al., 2012; Wang et al., 2016a). Drought-related damage to tea plants has grown more frequent and unexpected in recent years as a result of global climate change and increased water shortages. Consequently, it is critical to comprehend how the tea plant responds to water scarcity.

To cope with drought stress, the status of the morphology, physiology, biochemistry, and molecules need to be changed in tea plants. When the tea plants are exposed to drought stress, the relative water content (RWC) and water potential of tea leaves decrease but the soluble sugars in tea leaves increase (Panda and Upadhyaya, 2013; Zhou et al., 2014). Drought stress increases the levels of ROS and Malondialdehyde (MDA) in tea plants, causing oxidative damage and disrupting antioxidant mechanisms (Wang et al., 2016b). With molecular biology techniques, regulatory genes and the functional genes related to drought resistance have been identified in tea plants (Muoki et al., 2012), such as HSPs, LEAs, TFs, genes of antioxidant metabolism response, hormone metabolism-related genes, and sugar metabolism-related genes (Muoki et al., 2012; Zheng et al., 2016). Many plants might use their vast metabolic homeostasis to adapt to abiotic conditions by creating a variety of primary and secondary metabolites (Obata and Fernie, 2012). Photosynthesis, sugar synthesis, the tricarboxylic acid (TCA) cycle, glycolysis, amino acid, and hormone synthesis have all been implicated in plant drought stress response (Qian et al., 2018). Metabolomics has been extensively used in researching plant resistance to environmental changes, including drought stresses (Charlton et al., 2008). Even though extensive research has been done on tea plants under drought stress using transcriptomics or proteomics, there is little information on metabolite changes in tea leaves based on metabolomic technology under drought stress and re-watering.

We used metabolomics and lipidomics approaches to evaluate changes in tea leaf metabolites in response to drought stress. The degree of drought induction was classified into four phases based on leaf morphological changes: (i) normal irrigation was classified as CK, (ii) before wilting as drought stress 1(DS1), (iii) withering as drought stress 2 (DS2), and (iv) re-watering after drought as RW. The objective of this research was to determine the metabolic profiles of tea leaves under drought stress and to assess the resilience of tea plants to drought stress.

## Materials and methods

### Materials and treatments

The tea plant collection and plant growth were carried out with permission from the Qingdao Agricultural University. Experimental research using plants complied with institutional, national, or international guidelines. The tea plant cultivar (*C. sinensis* L. cv. Ruixue), a drought-resistant variety bred by our research group, was planted in the tea plantation of the Tea Research Institute, Qingdao Agricultural University in Shandong Province, China (36°19′N, 120°23′ E, 54.88 m above sea level). Two-year-old tea seedlings procured from the nursery of Qingdao Agricultural University were grown in pots filled with tea garden soils. The seedlings were acclimatized with regular irrigation for 10 days in the tea garden which was covered with a rain-shielding shelter. During the experiment (from 4 August to 1 September), the daily maximum temperature was 29.7°C, and the daily minimum temperature was 21°C. And the precipitation was 80.1 mm. By controlling the irrigated water volume to induce drought stresses on tea plants. Based on the morphological changes, the degree of drought induction was divided into four stages: (i) normal irrigation (600 mL water was irrigated into each pot every other day) called CK, (ii) before wilting (no irrigation for 5 days) called DS1, (iii) wilting (no irrigation for 10 days) called DS2 and (iv) re-watering after drought (600 mL water was re-irrigated into each pot in which tea plant was wilting and sampling was performed 3 days later) called RW. Measurements of water content in plant and soil were taken routinely from 8:00 am to 9:00 am. For the metabolomics experiments, two leaves of tea shoots at different stages were collected. Accordingly, the sample collection dates were 14, 19, and 29 August and 1 September 2014. The samples, each containing six biological replicates, were washed using distilled deionized water and divided into two parts. One part was immediately frozen using liquid nitrogen and stored at −80°C to analyze the metabolome and the other part was used for instant MDA measurement.

### Chemicals

Methanol and chloroform, which were used as extraction solvents, were purchased from Merck (Darmstadt, Germany) and Sigma–Aldrich (St. Louis, MO, United States), respectively. Ribitol, which was used as an internal standard for GC–MS, was purchased from Sigma-Aldrich. For the derivatization of metabolites, methoxyamine hydrochloride (Fluka, Buchs, Switzerland), N, O-bis (trimethylsilyl) trifluoroacetamide (Fluka), and pyridine (Sigma–Aldrich) were used. Alkane standard solutions of C8–C20 and C21–C40 n-alkane mixtures were purchased from FlukaChemika. The mobile phase consisted of 0.1% aqueous formic acid (A) and 0.1% formic acid in acetonitrile (B).

### Determinations of the water content in soil, relative water content, and Malondialdehyde index in tea leaves

The water contents of soil (SWC) were determined using a soil moisture measuring instrument (ZYN-TSW1, Beijing, China). The RWC of tea leaves was determined as described previously (Upadhyaya et al., 2013). The contents of MDA in tea leaves were measured as described by a previous study (Li et al., 2011). The absorbance levels of MDA were recorded at 532, 600, and 450 nm. The contents of MDA were calculated using the formula: MDA [μM] = 6.45*(A532 - A600) – 0.56*A450.

### Extraction procedure

#### Extraction procedure of GC-MS

The frozen tea sample was homogenized with a chilled pestle and mortar to a fine powder. A subsample was quickly transferred to a pre-weighed chilled 10 mL plastic Eppendorf tube using a precooled spatula and weighed to approx. 100 mg. A 1.4 mL aliquot of chilled methanol (−20°C) was added. This was followed by vortex mixing for 30 s. Then, 60 μL ribitol (0.2 mg/mL) internal standard was added before extraction and vortexed for 30 s. After centrifugation at 2,200 × g for 5 min, the sample was sonicated for 30 min. Then, 1.4 mL ddH_2_O (4°C) and 750 μL (−20°C) chloroform were added. The mixture was vortexed for 1 min. The extraction solution was centrifuged at 2,200 × g for 5 min to obtain a clear solution. Then, 1 mL of supernatant was transferred to a new 1.5 mL plastic Eppendorf tube. After centrifugation at 12,000 × g for 5 min, 800 μL of supernatant was transferred to a new 1.5 mL plastic Eppendorf tube and was evaporated using nitrogen. Carbonyl moieties were protected by methoximation, by adding 60 μL 15 mg/mL solution of the corresponding alkoxyamine hydrochloride in pyridine at room temperature for 16 h, and acidic protons were derivatized with 60 μL of N, O-bis (trimethylsilyl) trifluoroacetamide with 1% of trimethylchlorosilane for 1 h at room temperature. The tube was then centrifuged for 10 min at 12,000 × g. Retention indices were calibrated by the addition of C8–C20 and C21–C40 n-alkane mixtures to each sample.

#### Extraction procedure of LC-MS

The frozen samples from each accession were homogenized to a fine powder in liquid nitrogen with a chilled pestle and mortar. A subsample was quickly transferred to a 5 mL plastic Eppendorf tube using a spatula precooled in liquid nitrogen and weighed to ∼100 mg. A 1,000 μL aliquot of chilled methanol (−20°C) was added. This was followed by vortex-mixing for 30 s and transferred to an ultrasonic cleaner (70°C) for 15 min. Then, 500 μL chloroform and 1,000 μL ddH_2_O were added. The mixture was vortexed for 1 min and centrifuged for 15 min (4,000 × g). The supernatant was transferred to a new plastic Eppendorf tube and passed through a 0.2 μm filter membrane.

### GC-MS analysis

The derivatized sample (1 μL) was injected into an Agilent 7890A/5975C GC-MS system (Agilent, United States) for the profiling analysis and identification of important compounds using a 20:1 split injection ratio. A HP-5 MS capillary column (5% phenyl methyl silox: 30 m × 250 μm i.d., 0.25 μm; Agilent J&W scientific, Folsom, CA, United States) was used. The injection and interface temperatures were 280°C and 150°C, respectively, and the ion source was adjusted to 250°C. The temperature of the transfer line was maintained at 285°C. Separations were achieved using the following temperature program: 5 min isothermal heating at 40°C, followed by a 10°C per min oven ramp to 300°C, and a final isothermal heating at 300°C for 5 min. The linear velocity of carrier gas (helium) was constant at 1 mL/min.

Mass spectra were acquired using full-scan monitoring mode with a mass scan range of 50–500 m/z at a scan speed of 1,000 u/s. The ionization mode was electron impact at 70 eV, and the detector voltage was 0.9 kV (Shen et al., 2015).

### LC-MS analysis

Chromatographic separations were performed on a 100 × 2.1 mm ACQUITYTM 1.7 μm C18 column (Droughts Corp., Milford, CT, United States) using an ACQUITY Ultra Performance Liquid Chromatography system. The temperature of the sample manager was set at 4°C, and the injection volume was 4 μL for each analysis. The electrolytes 0.001 mM sodium formate and 15 mM ammonium acetate were added to the mobile phase A (water) and B (acetonitrile) for electrospray ionization (ESI) positive mode, and 15 mM ammonium hydroxide for negative-ion mode, respectively. Besides, 0.1% (V/V) formic acid was also added to the mobile phase. The linear gradient elution below was carried out at 0 min eluent B 50% (flow 0.3 mL per min); 0–5 min eluent B 50–60%; 5–6 min eluent B 60–80%; 6-12 min eluent B 80%–100%; 12-14 min eluent B 100%; 14-15 min eluent B 100–50%; and then column equilibration for 1 min.

The Xevo G2 instrument was operated in both positive and negative ESI modes; parameter settings for the measurement were as follows: source temperature (120°C), desolvation temperature (300 °C), positive ion capillary voltage (3,000 V), negative ion capillary voltage (2,500 V); sampling cone voltage (27 eV), extraction cone voltage (4 eV), collision energy (6 eV). Nitrogen used as desolvation and cone gas was at a flow rate of 650 and 50 L⋅h^–1^, respectively. Both full MS and the fragmentation mass spectra were acquired at a rate of 5 spectra⋅ s^–1^ in the range m/z 50-1,500. All analyses were obtained using leucine enkephalin for lock mass calibration to guarantee accuracy and reproducibility, at the concentration of 0.4 ng/L, in 50/50 of acetonitrile/H_2_O with 0.1% v/v formic acid.

### MS data processing and multivariate data analyses

Peaks with signal to noise ratios > 6 were extracted using an Agilent ChemStation. Unprocessed MS files were first converted into NetCDF format by the Agilent MSD Station and sequentially handled by XCMS software^1^. The relative quantification of each metabolite detected by GC-MS was calculated according to the peak area that normalized to the ribitol internal standard. Meanwhile, the relative quantification of each variable obtained from LC-MS was calculated according to the peak area normalized to the sum.

Multivariate statistics were performed using Soft Independent Modeling of Class Analogy-P version 11.0 (Umetrics AB, Umea, Sweden). All variables were unit variance scaled prior to orthogonal-partial least squares-discriminant analysis (OPLS-DA) for classification. A loading plot was employed to screen the notable metabolite differences in the OPLS-DA.

### Metabolite annotation

The commercially integrated database NIST08 and the publicly available database GOLM were used for annotating metabolites detected by GC-MS. The empirical formulas of possible metabolites detected by LC-MS/MS were firstly validated by the accurate molecular mass and were further verified by the Masslynx i-FIT algorithm. At last, the differential metabolites in tea leaves of different drought stresses or re-watered were confirmed by the accurate molecular mass, MS/MS fragments as well as the Human Metabolome Database (HMDB)^2^, Metlin^3^, LipidMaps^4^.

### Statistical analyses

Variables with variable importance in projection (VIP) > 1 that played important roles in the classification were selected for further analysis. Subsequently, an independent *t*-test was applied to exclude the variables without significant differences (*p* > 0.05) (GraphPad Prism 7.0). Hierarchical clustering analysis (HCA) was carried out using R software (version 3.0.3) to visualize and group metabolite profiles. The data were normalized based on the abundance of the internal standard and transformed with unit variance scaling (Shen et al., 2015).

## Results

### The phenotype, RWC, and MDA concentration changes of tea leaves under drought stress

**FIGURE 1:**
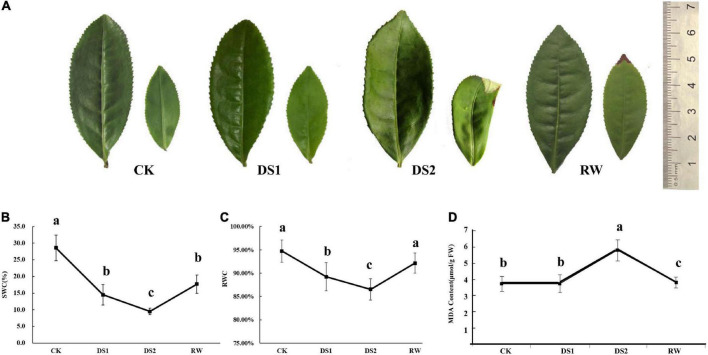
Phenotypic changes of mature and tender tea leaves **(A)**, the water content of the soil **(B)**, the relative water contents **(C)** and MDA contents **(D)** of tea leaves from tea seedlings under drought and re-water conditions.

To evaluate the instantaneous effects of drought stresses on tea plants, the leaf phenotype, RWC, and MDA concentration changes were recorded (Figure 1). The leaf phenotypes varied with the soil water contents (Figures 1A,B). As is shown in Figure 1A, the tea leaves displayed fully expanded green microphylls when the tea plants were normally watered. When the tea seedlings were exposed to the drought, the leaves became curled and crinkled and were seriously damaged with widespread wilting and the tender leaves were scalded to a degree (Figure 1A, DS2), which was an adaptive response to the damage caused by water losses, extreme temperatures, or combined stresses. The leaves recovered to their normal form after tea seedlings were rehydrated (Figure 1A, RW). The RWC of the leaves showed a decreasing trend during the drought treatments and increased after the seedlings were re-watered (Figure 1C). The Lipid peroxidation levels of the tea leaves under drought stress were expressed using the malondialdehyde (MDA) concentrations (Figure 1D). MDA contents of tea leaves significantly increased after the tea plants were exposed to severe drought stresses (DS2). However, when drought conditions were alleviated, the MDA contents in plants decreased and were lower than that in normal growth tea plants.

### Analysis of metabolites profiling detected by GC-MS

**FIGURE 2:**
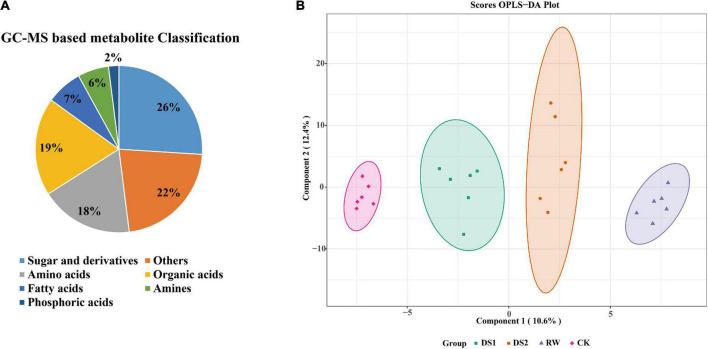
Pie chart of metabolite classification **(A)** and OPLS-DA score plots for metabolic profiling analysis **(B)**. The score of variation explained by each principal component is indicated on the axes. Each point corresponds to a plant sample, and different colors indicate the different treatments.

The final phenotype of plants exposed to the drought was influenced by the metabolites, which are directly involved in plant cell structure and metabolism. To figure out the metabolites critically affected by the drought, we first focused on the primary metabolites detected by GC-MS. In total, 288 metabolites were detected in tea leaves, 90 of which were annotated using the National Institute of Standards and Technology (NIST) and Wiley libraries (Supplementary Table 1). These metabolites were mainly sugars and derivates (24%), organic acids (19%), and amino acids (18%) (Figure 2A). Removing some of the metabolites lacking in most samples, a total of 78 metabolites were chosen and their concentrations were determined for further analysis.

To examine the metabolic changes in tea leaves in response to drought stresses and the significance of various metabolites contributing to these alterations, we analyzed the data using a supervised chemometric method, orthogonal-partial least squares-discriminant analysis (OPLS-DA) (Figure 2B). To assess the risk that the current OPLS-DA model was spurious, the permutation test for OPLS-DA was applied, and the results (for 200 permutations. R^2^Y = 0.992 and Q^2^ = 0.793, Supplementary Figure 1) indicated that the proposed OPLS-DA model was reliable. The OPLS-DA score plots exhibited an obvious separation among the four groups as the first principal component (PC1) and second principal component (PC2) accounted for 10.6 and 12.4%, respectively. And the four groups separated from each other. The major metabolites that contributed to the first two principal components were 5-methyl-pyrimidine, quinic acid, gulonic acid, 1,2,3-trishydroxybenzene, and linoleic acid etc.

### Analysis of metabolites profiling detected by HPLC-MS/MS

Lipids, as important molecules, are involved in energy storage, membrane components, and signaling (Perlikowski et al., 2016). To clarify how the lipid metabolites changed in response to the drought, we further analyzed the drought samples using HPLC-MS. A total of 5163 and 4835 clean retention time-exact mass pairs were obtained in the samples at the ESI (-) and ESI (+) ion modes, respectively.

**FIGURE 3:**
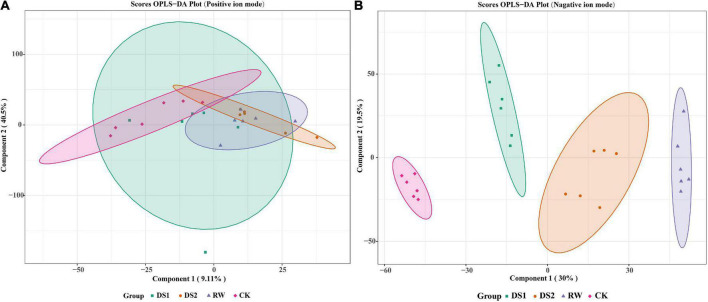
OPLS-DA score plots for metabolic profiling analysis in positive **(A)** and negative **(B)** ion modes. The score of variation explained by each principal component is indicated on the axes. Each point corresponds to a plant sample, and different colors indicate the different treatments.

To research the lipid composition changes in the membranes of tea leaves, the pre-processed LC-MS data were first multivariate statistically analyzed. The OPLS-DA score plot in the positive ion mode showed an indistinct separation among the four groups. However, the negative ion mode showed an obvious separation among the four groups, and the CK group was a bit remote from the other three groups (Figure 3). The first two components of the OPLS-DA score plot explained 9.11 and 40.5% of the variation in positive ion mode and 30 and 19.5% of the variation in negative ion mode, respectively (Figures 3A,B). The permutation test for OPLS-DA was applied, and the results (for 200 permutations. R^2^Y = 0.991 and Q^2^ = 0.948, Supplementary Figure 2) indicated that the proposed OPLS-DA model was reliable.

**FIGURE 4:**
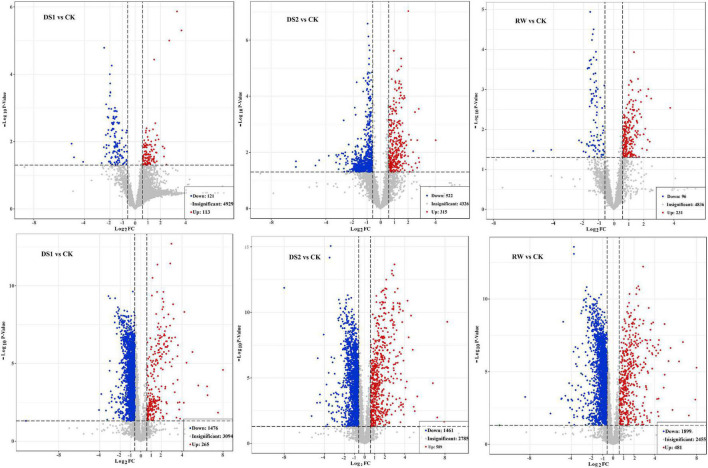
Volcano plot on LC-MS based metabolites that were significantly different in DS1 vs CK, DS2 vs CK, and RW vs CK comparisons in positive (upper) and negative (lower) ion modes, respectively. The red color indicates metabolites with fold change ≥ 1.5 and *p* value ≤ 0.05. The blue color indicates metabolites with fold change ≤ –1.5 and *p* value ≤ 0.05. The gray color indicates metabolites with no significant difference.

To find out the material basis of changes in the membranes of tea leaves, lipid metabolite changes of tea plants during the drought and re-watering process were studied. The obtained variants passing the VIP > 1 threshold in the OPLS-DA model and the *t*-test (*p* < 0.05) were considered significantly different metabolites between the two groups. There were 234, 837, and 327 differential ions between DS1 vs CK, DS2 vs CK, and RW vs CK comparisons in positive ion modes, respectively (Figure 4 upper). And there were 1741, 2050, and 2380 differential ions between DS1 vs CK, DS2 vs CK, and RW vs CK comparisons in negative ion modes, respectively (Figure 4 lower).

Then some significantly differential ions were randomly chosen and were further daughter ion scanned using Waters Q-TOF MSE mass spectrometry. The accurate mass with 30 ppm tolerance allowed possible elemental composition calculation of the empirical chemical formulas. And these ions were finally confirmed using HMDB^5^, LIPID MAPS (see text footnote 4), and METLIN^6^ to elucidate the putative ion structures (Supplementary Table 2). In the MSE collision mode, a lipid was defined only when the parent ion and daughter ion of an ion could be found simultaneously in the low-energy (6 eV) and high-energy (25 ev-60 eV) collision mode with the same retention time as a previous study described (Xu et al., 2010a; Li et al., 2015). Finally, 29 lipids, belonging to six different classes, phosphatidylglycerol (PG), phosphatidic acid (PA), sulfoquinovosyl-diacylglycerol (SQDG), phosphatidylcholine (PC), lyso-phosphatidylcholine (LysoPC), and phosphatidylinositol (PI), were identified (Supplementary Figure 3).

**FIGURE 5:**
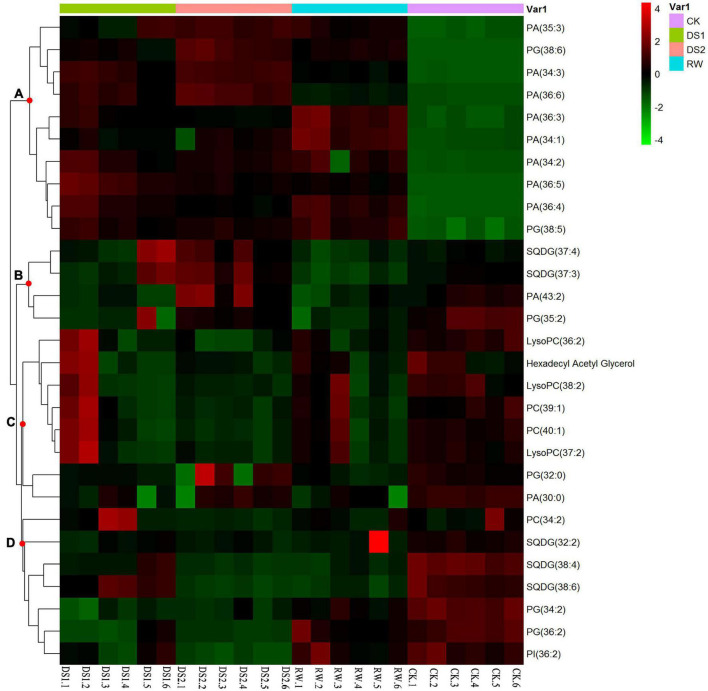
Hierarchical clustering of 29 lipids from tea leaf samples of four different conditions. Similarity assessment for clustering was based on the Euclidean distance coefficient and the average linkage method. Columns and rows represent individual metabolites and different samples, respectively. The levels of the metabolites changed and could be organized into four clusters, named **(A–D)**.

The identified lipids were also analyzed by hierarchical clustering. There was a clear separation of the lipids between the four kinds of leaves (Figure 5). The first cluster contained group A. The lipids were mainly PA and PG, and these metabolite levels increased when tea plants were exposed to drought or re-watered. The other cluster contained groups B, C, and D. Lipids in this group were mainly PC, LysoPC, and SQDG. The levels of these lipids decreased when tea plants were exposed to drought or re-watered conditions.

### The differential metabolites in tea leaves under DS1

Though more than 80 metabolites were identified in the tea samples, only 24 metabolites showed significant differences in DS1 compared with the regularly grown tea plants. These metabolites were monomethylphosphate, octadecatrienoic acid, sucrose, ethanolamine, and cyclohexane-1,3,5-trione identified by GC-MS (Table 1), and PA, PG, PI, and SQDG identified by LC-MS/MS (Table 2). Among the six GC-MS-detected differential metabolites, sucrose, ethanolamine, and cyclohexane-1,3,5-trione showed higher up-accumulation in tea leaves of DS1. As for the lipids, 8 PAs and 2 PGs were significantly up-accumulated with higher fold changes (log_2_(DS1/CK) > 2). The other 8 lipids including 2 SQDGs showed lower levels in the DS1 leaves (Table 2).

**TABLE 1:** Fold changes (FC) of significantly differential primary metabolites in leaves of *C. sinensis* cv. Ruixue in response to drought stress.

Metabolite name	DS1 / CK			DS2 / CK			RW / CK		
			
	VIP	FC	*P*-value	VIP	FC	*P*-value	VIP	FC	*P*-value
**Sugars and sugar alcohols**									
Sucrose	1.74	1.38*	0.050	1.82	1.92**	0.004	1.88	1.69**	0.001
Turanose	0.87	0.15	0.360	1.84	0.29*	0.031	0.63	0.16	0.441
Melezitose	1.35	***−0.32***	0.157	0.74	**−**0.31	0.335	1.52	−0.65*	0.018
Cellotriose	0.94	0.36	0.369	1.64	1.19*	0.013	1.28	***0.76***	0.067
Mannitol	0.13	0	0.985	1.05	***0.44***	0.161	1.34	0.38*	0.041
Myo-Inositol	1.65	***−0.56***	0.069	1.83	−0.93**	0.004	1.56	−0.47*	0.018
Inositol	0.54	0.05	0.778	1.652	0.42*	0.012	0.86	0.11	0.261
**Organic acids**									
Fumaric acid	1.11	**−**0.58	0.295	0.19	0.17	0.807	1.52	1.17*	0.016
Glyceric acid	0.82	**−**0.24	0.401	0.13	**−**0.04	0.874	1.77	0.76**	0.003
Glyoxylic acid	1.33	***−0.78***	0.197	1.4	−1.39*	0.045	0.44	**−**0.14	0.756
Quinic acid	0.66	**−**0.13	0.512	1.42	−0.40*	0.041	1.86	−0.59**	0.001
Malic acid	0.69	**−**0.22	0.49	0.65	**−**0.2	0.395	1.12	***0.30***	0.12
Succinic acid	0.92	0.22	0.361	1.2	***0.52***	0.095	1.78	0.70**	0.003
2,4,5-Trihydroxypentanoic acid	0.99	0.24	0.298	1.57	0.52*	0.020	1.56	0.35*	0.014
Linoleic acid	1.03	***−0.21***	0.276	1.52	−0.53*	0.026	1.26	***−0.23***	0.067
**Amino acids and Amines**									
Ethanolamine	1.76	2.34*	0.046	1.05	***1.89***	0.731	1.06	***0.04***	0.145
Pyroglutamic acid	0.36	0.16	0.745	1.57	2.08*	0.020	1.26	***1.71***	0.171
Glutamine	1.1	***0.65***	0.252	1.35	***1.17***	0.056	1.52	1.03*	0.016
**Others**									
1,2,3-Trishydroxybenzene	0.11	0	0.991	1.3	***−0.40***	0.067	1.43	−0.45*	0.028
5-methyl-Pyrimidine	0.62	–0.03	0.868	0.22	0.99	0.781	2.02	0.47**	0.000
Benzyl alcohol	3	***0.15***	0.945	1.15	***−0.68***	0.115	1.5	−1.22*	0.018
Cyclohexane-1,3,5-trione	2.01	1.58*	0.019	1.36	***1.6***	0.054	1.12	***0.8***	0.111
Galactosyl-glycerol	1.53	***0.37***	0.090	2.05	0.66**	0.000	1.65	0.69**	0.007
Monomethyl-phosphate	1.77	−0.74*	0.045	1.38	−0.98*	0.049	1.09	***−0.44***	0.110
Octadecatrienoic acid	1.87	0.79*	0.033	1.69	1.37**	0.010	1.36	0.70*	0.050

The fold changes were log_2_(DS1/CK), log_2_(DS2/CK), log_2_(RW/CK), respectively. The bold italic indicates metabolites were VIP > 1, *means *p* < 0.05; **means *p* < 0.01. Only the metabolites in bold italic style and * or ** marked were considered significantly differential metabolites.

**TABLE 2:** Fold changes (FC) of significantly differential lipids in leaves of *C. sinensis* cv. Ruixue in response to drought stress.

Metabolite name	DS1 / CK			DS2 / CK			RW / CK		
			
	VIP	FC	*P*-value	VIP	FC	*P*-value	VIP	FC	*P*-value
PA(35:3)	1.25	2.37**	0.000	1.41	2.88**	0.000	1.32	2.58**	0.000
PG(38:6)	1.42	2.34**	0.000	1.4	2.93**	0.000	1.34	2.52**	0.000
PA(34:3)	1.39	2.71**	0.000	1.42	2.97**	0.000	1.34	2.25**	0.000
PA(36:6)	1.4	2.56**	0.000	1.41	2.91**	0.000	1.34	1.72**	0.000
PA(36:3)	1.38	3.56**	0.000	1.41	3.18**	0.000	1.3	4.14**	0.000
PA(34:1)	*1.4*	3.61**	0.000	1.16	3.69**	0.001	1.3	4.54**	0.000
PA(34:2)	1.35	2.99**	0.000	1.42	2.87**	0.000	1.06	2.82**	0.002
PA(36:5)	1.41	2.53**	0.000	1.41	2.10**	0.000	1.34	2.04**	0.000
PA(36:4)	1.41	2.45**	0.000	1.41	2.02**	0.000	1.33	2.49**	0.000
PG(38:5)	1.4	2.62**	0.000	1.4	2.60**	0.000	1.32	2.80**	0.000
SQDG(37:4)	0.47	0.4	0.311	1.01	0.49**	0.010	1.02	−0.37**	0.004
SQDG(37:3)	0.15	0.06	0.743	0.99	0.33*	0.012	1.23	−0.40**	0.000
PA(43:2)	1.25	−0.48**	0.000	0.66	*0.28*	0.131	1.03	−0.47**	0.004
PG(35:2)	0.85	**−**0.98*	0.047	1.01	−0.44**	0.010	1.18	−1.43**	0.000
LysoPC(36:2)	0.22	**−**0.22	0.687	2.04	−1.83**	0.000	2.25	−0.87**	0.014
Hexadecyl Acetyl Glycerol	0.01	**−**0.18	0.739	1.41	−0.70*	0.044	1.23	***−0.43***	0.228
LysoPC(38:2)	0.57	**−**0.41	0.405	1.97	−1.00**	0.001	1.15	***−0.43***	0.266
PC(39:1)	0.1	**−**0.09	0.841	2.04	−0.89**	0.000	0.91	**−**0.3	0.382
PC(40:1)	0.06	**−**0.09	0.841	2.32	−0.96**	0.000	1.27	***−0.35***	0.216
LysoPC(37:2)	0.11	**−**0.02	0.964	2.15	−0.90**	0.000	1.27	***−0.4***	0.216
PG(32:0)	1.29	−−0.52**	0.000	0	0	0.996	1.14	−0.60**	0.000
PA(30:0)	1.05	-0.76**	0.008	0.62	*-0.37*	0.158	1.01	−0.81**	0.005
PC(34:2)	0.02	0.49	0.565	1.4	−2.34*	0.048	0.86	**−**0.6	0.412
SQDG(32:2)	1.22	−0.51**	0.001	1.32	−0.60**	0.000	0.02	**−**0.02	0.967
SQDG(38:4)	1.26	−0.47**	0.000	1.4	−0.80**	0.000	1.31	−0.72**	0.000
SQDG(38:6)	0.38	**−**0.11	0.418	1.37	−1.19**	0.000	1.27	−1.04**	0.000
PG(34:2)	1.4	−0.68**	0.000	1.37	−0.56**	0.000	1.24	−0.33**	0.000
PG(36:2)	1.28	−0.78**	0.000	1.39	−0.86**	0.000	0.75	–0.23	0.062
PI(36:2)	1.06	−0.37**	0.008	1.36	−0.70**	0.000	0.41	–0.1	0.339

The fold changes were log_2_(DS1/CK), log_2_(DS2/CK), log_2_(RW/CK), respectively. The Bold italic indicates metabolites were VIP > 1, *means *p* < 0.05; **means *p* < 0.01. Only the metabolites in bold italic style and * or ** marked were considered significantly differential metabolites.

### The differential metabolites in tea leaves under DS2

With the intensification of drought stress, the metabolites in tea leaves of DS2 occurred more complex changes than that of DS1 to cope with the adversity. Metabolites belonging to various classes showed significant changes to cope with the severe drought stress (Tables 1, 2). For example, the pyroglutamic acid, derived from glutamine and glutamic acid, showed a significant increase and the log_2_(DS2/CK) was 2.08. Sucrose, a signaling molecule coordinating the relationship between plant sources and sinks, also increased significantly with the log_2_(DS2/CK) = 1.92. Besides, cellotriose and fatty acid octadecatrienoic acid also up-accumulated significantly in the tea leaves under DS2 with log_2_FC bigger than 1, but smaller than 1.5. Meanwhile, glyoxylic acid was found down-accumulated in the tea leaves of DS2 and log_2_(DS2/CK) was –1.387. Monomethylphosphate and myo-Inositol also decreased in the DS2-leaves and the log_2_(DS2/CK) were both about –0.9. Quinic acid, a secondary metabolite related to the shikimic acid pathway, decreased in DS2 tea leaves with the smaller |log_2_FC|. Those PAs and PGs which were significantly up-accumulated in tea leaves of DS1 were also found to increase in the tea leaves under DS2 with higher fold changes (log_2_ (DS2/CK)>2). We also found that several PC and LysoPC detected by the LC-MS/MS pos mode decreased in the tea leaves under severe drought stress. However, these PC and LysoPC types lipids did not present significant differences in DS1 tea leaves.

### The differential metabolites in tea leaves under RW

When the tea plants suffered from drought stress were re-watered, the damage to tea plants caused by drought was alleviated to some extent. The responses after rewatering were generally different from those observed at DS2, with the metabolites presenting significantly different levels between rewatering and drought stress or normal growth over time. Despite the accumulations of metabolites during recovery, they were still significantly lower in re-watered leaves compared with those in the well-watered control samples. Except for sucrose, fumaric acid derived from the TCA cycle and glutamine were also significantly increased, and the log_2_(RW/CK) of them were 1.17 and 1.03, respectively. The metabolites including benzyl alcohol, 1,2,3-trishydroxybenzene, myo-inositol, and quinic acid decreased in this stage. These metabolites accumulated at higher levels in the CK-leaves compared with that in the RW-leaves. The contents of PA (34:1) and PA (36:3) lipids still increased in the leaves after the tea plants were re-watered, and the log_2_(RW/CK) was bigger than 4. The other PAs (34:2, 34:3, 36:6, 36:5, 36:4, 35:3) and PGs (38:5, 38:6) also accumulated at higher levels in the RW leaves with the log_2_(RW/CK)>2. In addition, 3 SQDGs were less accumulated in the RW-leaves. To our interest, except LysoPC (36:2) [log_2_(RW/CK) = -0.87], the PC and LysoPC were not differential in the RW-leaves.

### Analysis of metabolite correlations in tea leaves under different treatments

**FIGURE 6:**
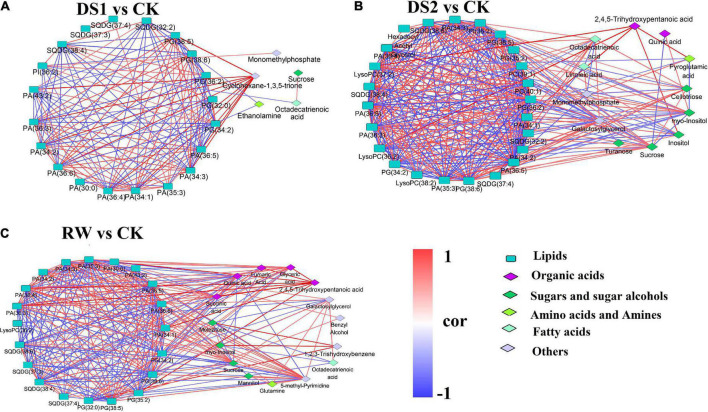
Network map of correlation analysis among differential metabolites: **(A)** DS1 vs. CK; **(B)** DS2 vs. CK; **(C)** RW vs. CK. Metabolites represented by rounded rectangles were lipids. Metabolites represented by diamond were primary metabolites detected by GC-MS. The same color indicates metabolites in the same metabolic function group. Correlations are shown by connected lines. Red lines are positive correlations, and blue lines are negative correlations.

To investigate the lipid–primary metabolites correlation in tea leaves responding to the drought stress, the values of Pearson’s pairwise correlation coefficients for each pair of the differential metabolites in DS1, DS2, and RW were calculated. The correlations with r^2^ ≥ 0.49 and *p* value < 0.05 were chosen as significant correlations (Figure 6).

In the DS1 vs CK comparison, 140 significant correlations were found, including 69 positive and 71 negative correlations (Figure 6A). However, only 14 correlations were found between lipid and primary metabolites. Cyclohexane-1,3,5-trione was positively correlated with PA (36:6, 36:5, 36:4, 36:3, 34:3, 34:2, 34:1) and PG (38:5). Ethanolamine was negatively correlated with SQDG (32:2) and PA (30:0).

In the DS2 vs CK comparison, 323 correlations were found, including 164 positive and 159 negative correlations (Figure 6B). This revealed that more metabolites are highly associated within the network, especially amongst the lipids. In total, 45 correlations were found between lipids and primary metabolites, and the primary metabolites was dominated by sugar and sugar alcohols. Octadecatrienoic acid was positively correlated with SQDG (37:4), PG (38:6) and PA (36:6, 36:5, 36:4, 36:3, 35:3, 34:3). Cellotriose was positively correlated with sucrose, galactosylglycerol, octadecatrienoic acid, SQDG (37:4), PG (38:6) and PA (36:6, 36:4, 36:3, 34:3). Galactosylglycerol was positively correlated with octadecatrienoic acid, turanose, sucrose, SQDG (37:4), PG (38:6, 38:5) and PA (36:6, 36:5, 36:4, 36:3, 35:3, 34:3, 34:2). Whereas, Galactosylglycerol was negatively correlated with myo-inositol, PG (36:2, 34:2), PI (36:2), SQDG (38:6, 38:4, 32:2), LysoPC (37:2, 36:2) and PC (40:1). Inositol showed negative correlation with PI (36:2), PG (34:2), PC (39:1, 40:1) and LysoPC (37:2, 36:2).

In the RW vs CK comparison, 282 correlations were found, including 143 positive and 139 negative correlations (Figure 6C). 85 correlations were found between lipids and primary metabolites, and the primary metabolites were dominated by organic acids, sugar, and sugar alcohols. Quinic acid showed a positive correlation with SQDG (38:6, 38:4). Glyceric acid showed a positive correlation with succinic acid, PG (38:6, 38:5), and PA (36:6, 36:5, 36:4, 36:3, 35:3, 34:3, 34:1). Fumaric Acid was positively correlated with succinic acid, glyceric acid and PA (35:3, 34:1). 2,4,5-Trihydroxypentanoic acid showed a positive correlation with succinic acid, glyceric acid, fumaric Acid, and PA (36:6, 36:4, 36:3, 35:3, 34:1). Melezitose was positively correlated with myo-inositol, PG (32:0) and SQDG (38:6), while was negatively correlated with sucrose and octadecatrienoic acid. Galactosylglycerol was positively correlated with sucrose, glutamine, octadecatrienoic acid, PG (38:6), and PA (36:6, 34:3), while was negatively correlated with melezitose and LysoPC (36:2). 5-methyl-Pyrimidine was positively correlated with mannitol, glutamine, sucrose, and PAs, while it was negatively correlated with PGs and SQDGs. These results suggested that tea plants mobilized more complicated metabolites interactions to cooperate to deal with severe drought damage.

## Discussion

Drought stress is a major environmental condition that restricts crop growth, development, and yield in arid and semi-arid regions. To cope with the detrimental drought stress, plants undergo a variety of morphological, physiological, molecular, and biochemical changes, in which small molecules, genes, and proteins play a prominent role (Michaletti et al., 2018). Therefore, the plant stress response is a complex dynamic process designed to establish new homeostasis under adverse growth conditions. And the changes in leaf morphological and physiological traits could more accurately indicate the degree of stress (Sperdouli and Moustakas, 2012). To investigate metabolomic mechanisms of tea plants responding to water deficiencies and re-watering, tea seedlings were subjected to medium-term water stress followed by a short-term re-supply of water. The various phenotypes of tea leaves might signify metabolite modifications in tea leaves under distinct drought conditions (Figure 1), because the final phenotypes of a plant influenced by metabolites which were involved in plant cell structure and metabolism (Krasensky and Jonak, 2012). RWC results indicated that the tea plants suffered from the drought stress under DS1. However, the MDA contents in tea leaves under DS1 showed no significant changes suggesting that tea plants augmented a few metabolites such as sucrose and ethanolamine to maintain cell homeostasis and prevent oxidative damage to cell membranes. When the tea plants were exposed to DS2, significant increases were found in the content of MDA. This result indicated a significant amount of oxidative damage to lipid membranes in tea leaves, and the tea plant had a severe damage phenotype. However, once the tea plants were re-watered, the MDA levels were lower than the CK levels, and the tea leaves were no longer wilted; in fact, the tender leaves were practically restored to normal. Metabolic adjustment in response to drought was a dynamic and multifaceted process that depended on the strength and duration of the drought. In our findings, drought stress had a significant impact on the distributions of water-soluble metabolites and lipids in tea leaves.

### More sugars and sugar alcohols were induced to cope with DS2

**FIGURE 7:**
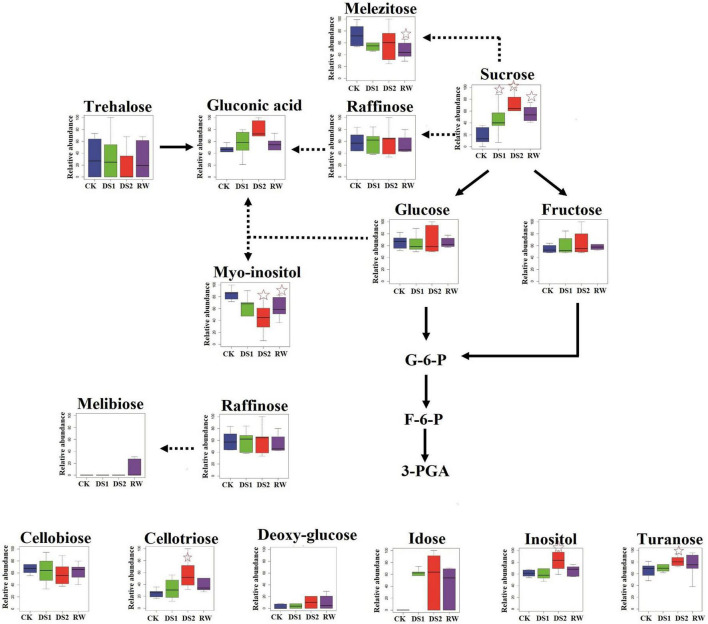
Differences in sugar and sugar alcohol metabolisms among tea plants of different drought stresses and re-watering. The *x*-axis represented the treatments. The *y*-axis box plots indicated the scaled intensity median (—) values: top/bottom ranges of boxes indicated upper/lower quartiles, respectively; top/bottom whiskers indicated the maximum/minimum distribution of the data. The five-pointed star indicated that the metabolite was significantly differential in the sample compared with CK.

Sugars and sugar alcohols, including sucrose, mannitol, trehalose, and turanose, inositol are among the best-studied metabolites in resurrection plants, and they are reported to accumulate in vegetative tissues in response to drought stress (Todaka et al., 2017). In the present study, sucrose content was found to increase when the tea plants were exposed to the increasing drought stresses. When the tea plants were re-watered, the sucrose content in the RW-leaves was lower than in the DS2-leaves but greater than in the CK-leaves (Figure 7). In a recent work, the results disclosed that the sucrose synthesis-related gene SPS (sucrose-phosphate synthase) was upregulated in tea during drought stress but downregulation was found under recovery conditions (Liu et al., 2016), which verified our results from the gene expression perspective. T. kotschyanus (Du et al., 2020) showed a strong negative connection between RWC and sucrose in a prior investigation. We also looked at their association in our study on tea leaves, and the results indicated that the two indices were not substantially connected with each other (PC = 0.138). Therefore, the increase of sucrose did not directly increase the RWC of tea leaves in this experiment. Research has shown that many plants evolved a mechanism in response to drought stress called osmotic adjustment, which means the accumulation of osmolyte to reduce the osmotic potential and finally maintain cell turgor and plant metabolic activity (Patakas et al., 2002; Zeng et al., 2015). Sucrose, fructose, and glucose are important osmolyte in the osmotic adjustment. In the present research, we also found that fructose and glucose levels varied in response to drought stress, but not significantly (Figure 7). These finding might imply that sucrose played an important role in tea plants’ osmotic adjustment while they were subjected to drought stress. In totally desiccation-tolerant mosses, sucrose was the only free sugar available for cellular protection (Nakajima et al., 1992; Toldi et al., 2009). The accumulation of sucrose occurred relatively in the late period after dehydration. It is usually initiated at a relative water content of less than 60%, but the majority occurs at 20% (Toldi et al., 2009). The consensus is that the accumulation of sugars is one of the last preparatory steps in the cell protection pathway when the cell is fully committed to a period of quiescence. Thus, we speculated that sucrose was a sugar that rapidly responded to drought, and the gradual accumulation also could prevent membrane fusion after wilting.

As the drought stress became severe, some other sugars and sugar alcohols were also induced and accumulated in DS2 leaves, including cellotriose, turanose, galactosylglycerol, and inositol (Figure 7). Cellooligomers (COMs) including cellotriose were identified as novel chemical mediators in *Arabidopsis thaliana*, which inform the cell about the alterations in and around the cell wall (Ralf, 2018). COMs initiated Ca^2+^-dependent signaling events, including reprograming the cell and adjusting the expression and metabolite profiles as well as activating innate immunity responding to changes in their rhizosphere environment and the state of the cell wall (Ralf, 2018). Cellotriose was reported more active in elevating [Ca^2+^]cyt in the roots of *Arabidopsis* than other COMs (Johnson et al., 2014). Calcium had the ability to alleviate the adverse effects of drought stress on plants and participated in the process of anti-drought response signaling (Upadhyaya et al., 2011). Accordingly, we speculated that cellotriose might execute the signaling-inducing functions and initiate the Ca^2+^-dependent signaling in tea plants. The increase of cellotriose might promote its synergistic reaction with other signals and activate the local and systemic responses to cope with the severe drought stress. And when the stress got attenuated (at RW-stage), the reaction dominant by cellotriose would also decrease, so the cellotriose intensity decreased in tea leaves after they were rewatered.

Sugar alcohols had been reported to mimic the water structure and could maintain an artificial sphere of hydration around macromolecules, as they contain water-like hydroxyl groups (Pereira et al., 2014). Interestingly, the myo-inositol, a cyclic polyol, was down-accumulated in drought-exposed tea plants, which was different from reports in chickpea (Çevik et al., 2014), maize (Obata et al., 2015) and olive trees (Mechri et al., 2015). In these plants myo-inositol accumulated when they were exposed to drought stress. As a ubiquitous plant metabolite, myo-inositol played an important role in almost all biological systems. It could be synthesized into many molecules, such as phosphatidylinositol, raffinose family members, and ascorbic acid (AsA) (Song et al., 2016). Myo-inositol also had the function of signaling transduction, cell wall formation, phosphate storage, osmotic pressure regulation, and antioxidation (Donahue et al., 2010; Song et al., 2016). It was considered that the increase in myo-inositol in the aforementioned plants imposed to drought stress could augment the osmotic potential of the cell, thus balancing the osmotic potential of an externally increased osmotic pressure (Donahue et al., 2010). In tea plants, myo-inositol may also help to balance the osmotic potential. Its level decreased may because it was used to produce ascorbic acid, a major plant antioxidant that aids in the counterbalancing of any oxidative damage. In the future, this theory should be tested.

### Lipids remodeled under drought stress and recovery

The maintenance of membrane integrity and stability under drought stress is a major factor for drought tolerance in plants (Bajji et al., 2002). Membrane lipids, which are the main targets for environmental stresses, play important roles in membrane stabilization under adverse conditions (Torres-Franklin et al., 2007; Wang et al., 2020). Lipidome adjustments and membrane lipid remodeling are two strategies used mostly by plant cells to adapt against abiotic stressors such as drought (Moradi et al., 2017). Plants gradually developed the ability to positively alter lipid composition to adapt to adverse conditions. In this study, the level of the lipid metabolite PA (34:1, 34:2, 34:3, 35:3, 36:3, 36:4, 36:5, 36:6) increased statistically (*p* < 0.01; Supplementary Figure 4 and Table 2) in tea leaves under drought stress, which was consistent with the previous study showing a rapid and transient increase of PA levels in response to drought in plants (Testerink et al., 2007). PA is a signaling lipid (Testerink and Munnik, 2011), and its formation can give cells spatial and transitory information about their surroundings by participating as a protein-docking site in cellular membranes (Testerink and Munnik, 2005). PA could inhibit the activity of ABI1, a PP2C-type protein phosphatase that negatively regulated ABA responses (Testerink and Munnik, 2011). As a result, an increment in PA in tea plants under drought may positively modulate ABA responses, enhancing drought tolerance in tea plants. PA was involved in the synthesis of other membrane lipid components in plants, in contrast to being a crucial signaling molecule. PA could be converted to diglyceride, or diacylglycerol (DAG) and CMP-PA, promoting the synthesis of PG, PC, and PI to complement their degradation by activated phospholipases and galactolipase under drought stress (Zhai et al., 2013; Kolesnikov et al., 2022). In our work, we observed a substantial decrease (*p* < 0.01) in every molecular species of PG (34:2, 36:2), SQDG (32:2, 38:4, 38:6), PC (39:1, 34:2, 40:1), LysoPC (36:2, 38:2, 37:2), and PI (36:2) in tea leaves response to drought stress, which is consistent with previous reports on the other plant species (Lu et al., 2012). PC, PE, and PG were reported as primary degradation targets under drought stress (Wang et al., 2020). Therefore, we guessed that the decrease in PG and PC might be because their degradation was stronger than their synthesis. These findings reveal that during drought stress, tea plants modify membrane fluidity by altering lipid unsaturation indices in cellular compartments.

## Conclusion

**FIGURE 8:**
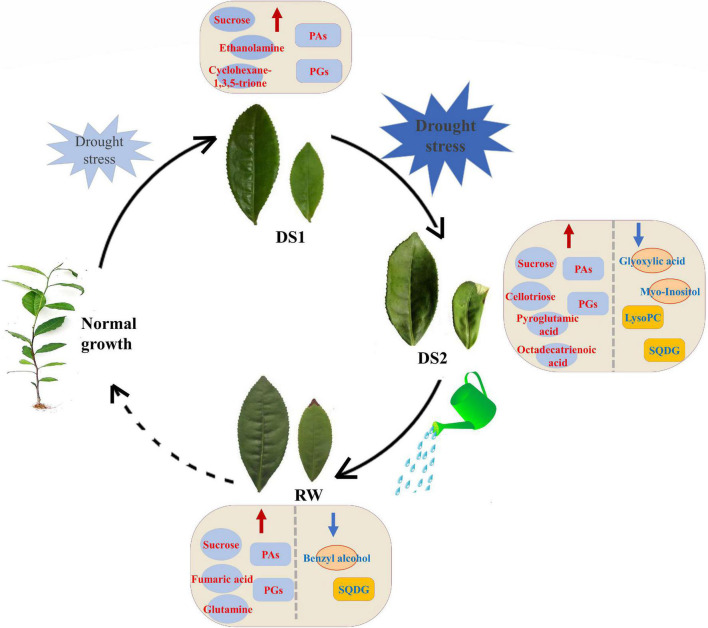
Models of possible mechanisms of tea plants responding to different water conditions. The red and blue arrows indicated that the levels of metabolites increased and decreased, respectively.

Drought stress has a considerable influence on primary and lipid metabolism in tea plants, according to the findings reported here. During drought and subsequent re-watering periods, the abundance of both primary and lipid metabolite species altered (Figure 8). Drought stress, especially severe ones, evoked the accumulation of sugars, sugar alcohols, and PAs, including sucrose, cellotriose, turanose, and different PA species (35:3; 34:3; 36:6; 36:3; 34:1; 34:2; 36:5; 36:4). Conversely, the organic acids, and the PG, SQDG, PC, LysoPC, and PI, decreased significantly in tea leaves under drought stresses. Furthermore, metabolic recovery was only limited, with the majority of the investigated metabolites having significantly different levels in re-watered tea plants compared to well-watered tea plants. Variations in these metabolite levels, as well as those of their precursors, might be considered as a method for adapting to drought conditions. The findings showed that comprehensive metabolomic and lipidomic approaches were useful in elucidating the impact of drought stress on tea plant metabolism. Our findings are valuable for understanding the mechanisms underlying drought tolerance in tea plants from a metabolic perspective, as well as utilizing these substances to increase drought tolerance in tea plants for better growth development and yield.

## Data availability statement

The original contributions presented in this study are included in the article/Supplementary material, further inquiries can be directed to the corresponding author.

## Author contributions

JS and SW collected and organized data and wrote the manuscript. LS helped to do the experiment and collect and organize data. YW and KF participated in designing the experiment and directed the study. CL carried out the experiment and helped to collect and organize data. HW reviewed the manuscript. CB and FZ reviewed the manuscript and gave useful advice. ZD raised the hypothesis underlying this work, designed the experiment, and helped to organize the manuscript structure. All authors contributed to the article and approved the submitted version.

## Footnotes

1
www.bioconductor.org/

2
https://hmdb.ca/metabolites

3
http://metlin.scripps.edu

4
http://www.lipidmaps.org

5
http://www.hmdb.ca

6
http://metlin.scripps.edu
