# Supplementary material for: Dynamic changes in metabolic and lipidomic profiles of tea plants during drought stress and re-watering
Source: Front Plant Sci. 2022 Sep 2;13:978531. doi: 10.3389/fpls.2022.978531 (PMC9478477; doi:10.3389/fpls.2022.978531)
Supplement: Supplementary file 1 [file Table_1.DOCX]

Supplementary Material

# Supplementary Figures and Tables

## Supplementary Figures


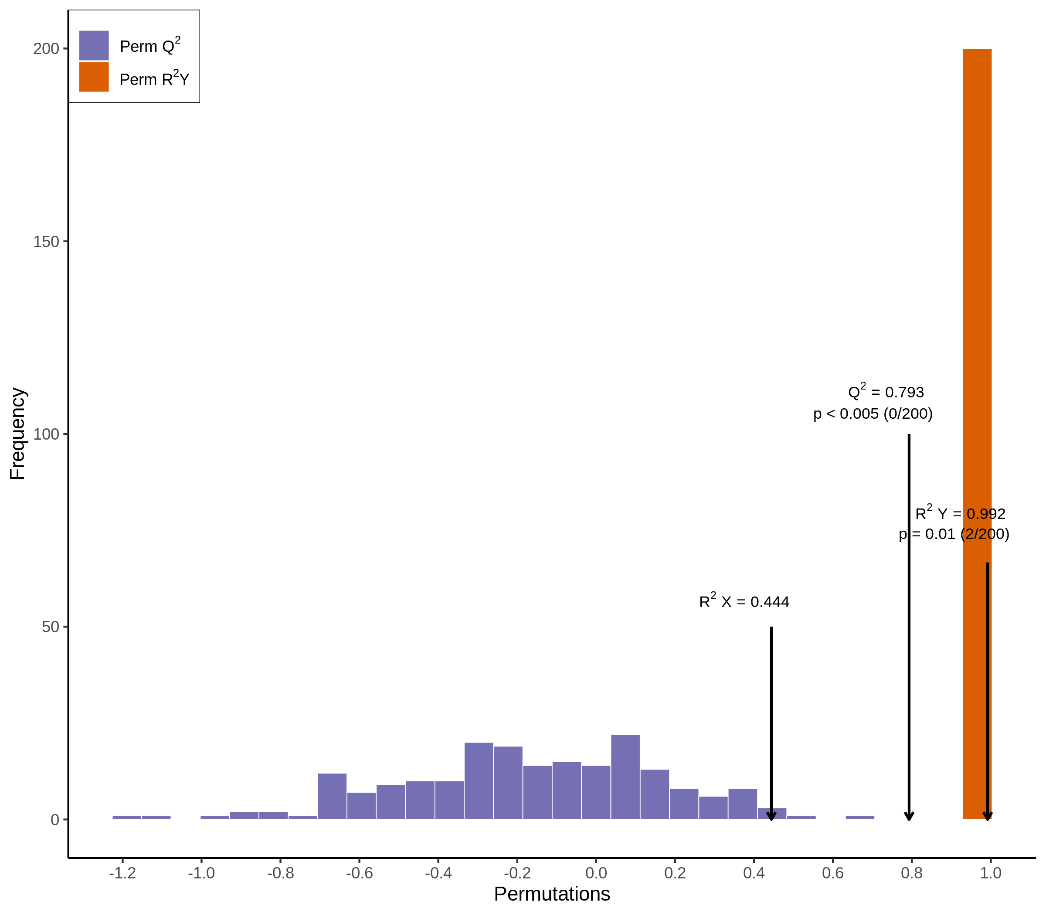


**Supplementary Figure1** Validation of OPLS-DA model based on the GC-MS data.


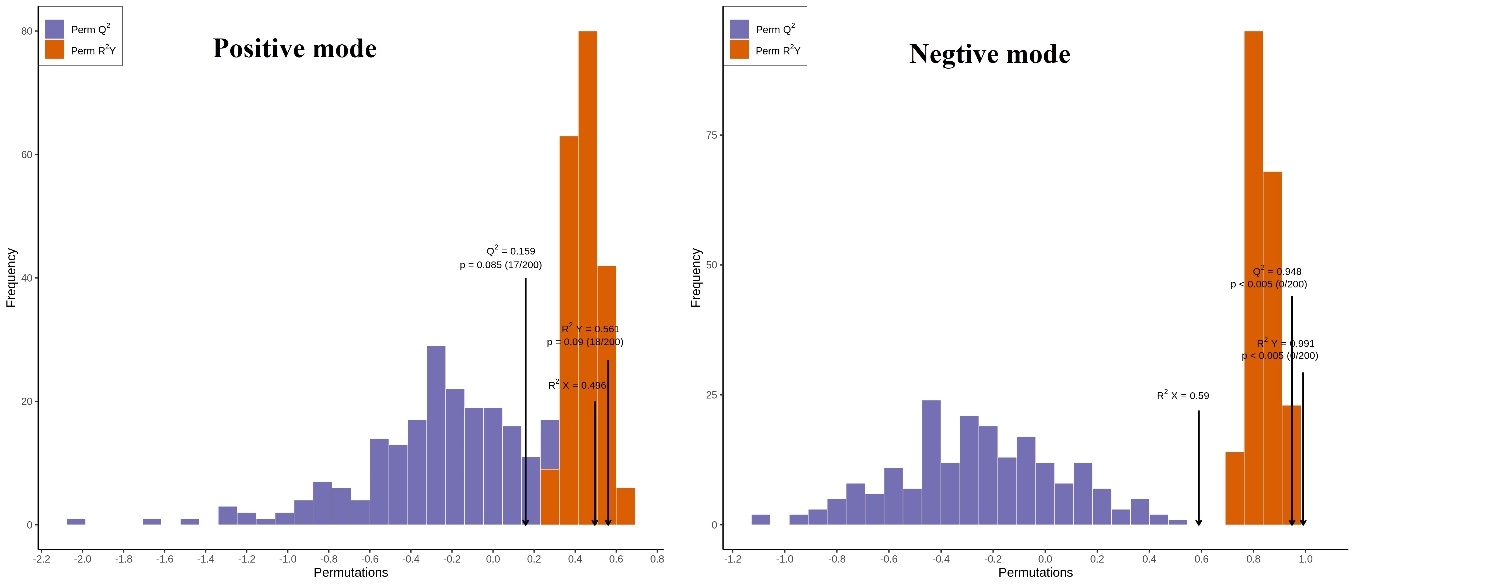


**Supplementary Figure 2** Validation of OPLS-DA models based on the LC-MS data acquired at the positive and negative modes.


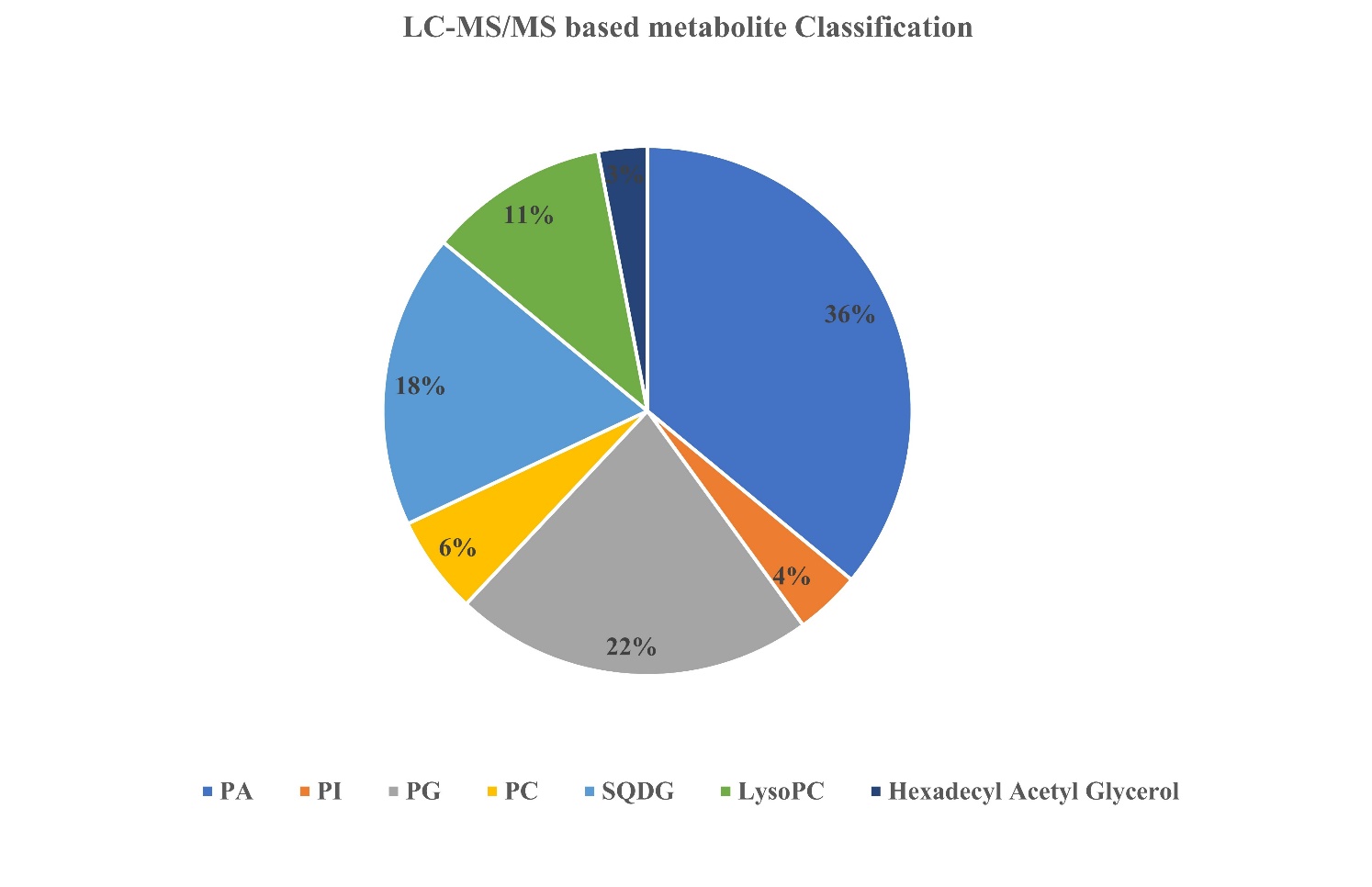


**Supplementary Figure 3** Categorization of 29 identified lipid metabolites detected in tea leaves under drought stresses and re-watering.


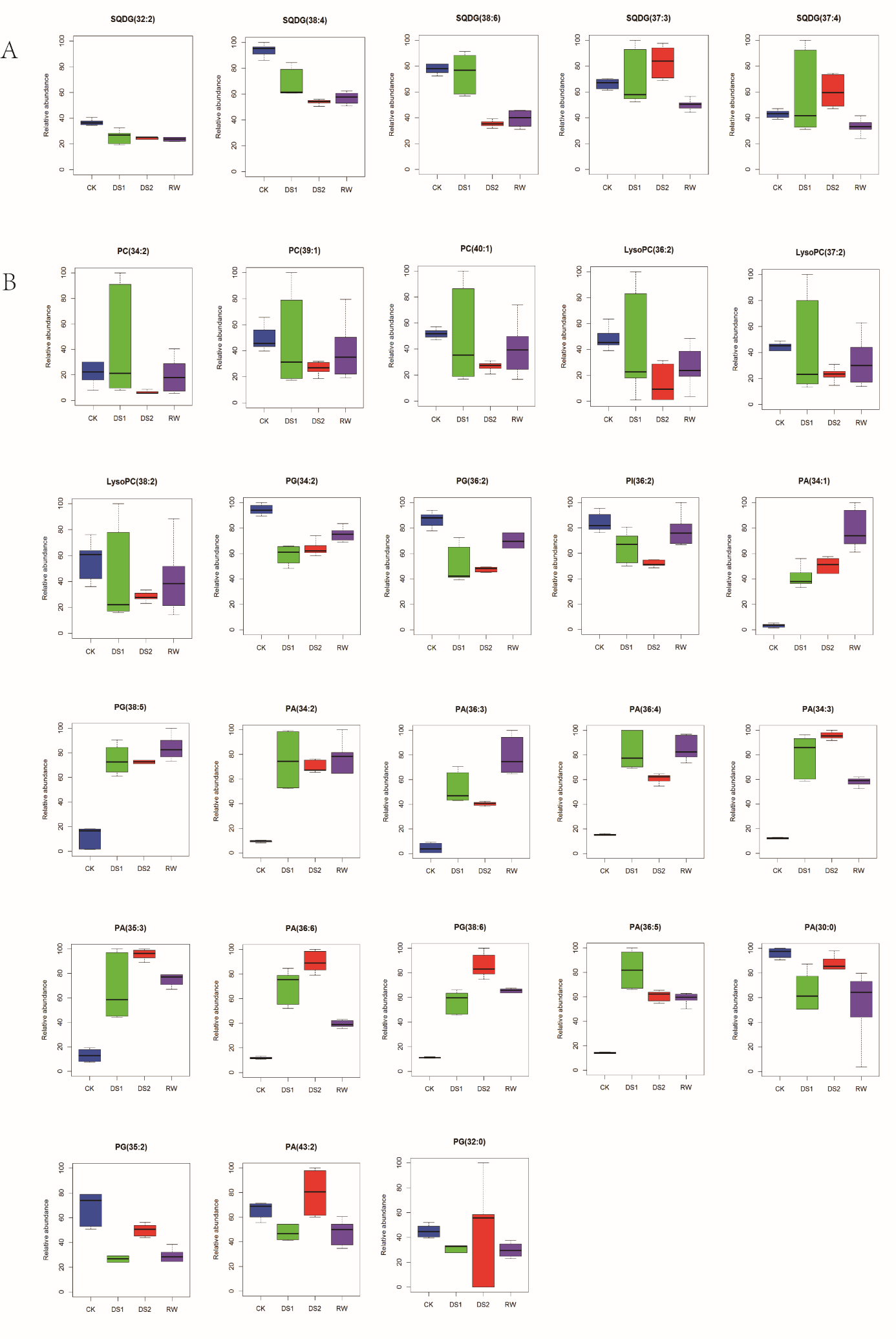


**Supplementary Figure 4** Boxplots of representative metabolites of (A) photosynthetic and (B) structural lipids. The x-axis represented the treatments. The y-axis box plots indicated the scaled intensity median (—) values: top/bottom ranges of boxes indicated upper/lower quartiles, respectively; top/bottom whiskers indicated the maximum/minimum distribution of the data.

## Supplementary Tables

## Supplementary Table 1 The m/z, retention time and detection platform of the identified metabolites.

| ID | m/z | RT | Detection Platform |
| --- | --- | --- | --- |
| 1,2,3-Trihydroxybenzene | 342.2 | 19.032 | GC-MS |
| 1,4-bimethylethyl-Benzene | 175.2 | 15.113 | GC-MS |
| 2,4,5-Trihydroxypentanoic acid | 245.1 | 20.324 | GC-MS |
| 2-Ketomalonic Acid | 276.1 | 17.166 | GC-MS |
| 3,4,5-Trihydoxybenzoic Acid | 458.3 | 23.557 | GC-MS |
| 3,4-Dihydroxymandelic Acid | 355.1 | 24.483 | GC-MS |
| 3-Hydroxypyridine | 152.1 | 11.555 | GC-MS |
| 3-Methylene-1,4-Butanediol | 143.1 | 15.155 | GC-MS |
| 4-Aminobutyric acid | 304.2 | 18.831 | GC-MS |
| 5-methyl-Pyrimidine | 255.1 | 18.056 | GC-MS |
| Acetamide | 188.1 | 7.809 | GC-MS |
| Alanine | 116.1 | 12.806 | GC-MS |
| ALPHA-Glycerophosphorylglycerol | 357.1 | 25.858 | GC-MS |
| Asparagine | 231.0 | 20.542 | GC-MS |
| Aspartic acid | 232.1 | 18.165 | GC-MS |
| Benzyl Alcohol | 165.1 | 13.581 | GC-MS |
| BETA-Alanine | 248.1 | 17.536 | GC-MS |
| Caffeine | 194.1 | 22.457 | GC-MS |
| Carbodiimide | 171.1 | 10.252 | GC-MS |
| Catechine | 368.2 | 31.772 | GC-MS |
| Cellobiose | 399.4 | 30.449 | GC-MS |
| Cellotriose | 204.1 | 28.000 | GC-MS |
| Citric acid | 273.1 | 22.200 | GC-MS |
| Cyclohexane-1,3,5-trione | 392.2 | 21.632 | GC-MS |
| Cystathionine | 392.1 | 14.469 | GC-MS |
| deoxy-glucose | 276.1 | 21.826 | GC-MS |
| Dodecamethylpentasiloxane | 281.1 | 14.014 | GC-MS |
| Eicosanoic acid | 349.2 | 27.592 | GC-MS |
| Ethanolamine | 174.1 | 15.348 | GC-MS |
| Ethylamin | 130.1 | 9.741 | GC-MS |
| Fructose | 319.2 | 23.114 | GC-MS |
| Fumaric Acid | 245.1 | 16.413 | GC-MS |
| Galactosylglycerol | 337.2 | 26.933 | GC-MS |
| Glucaric acid | 333.2 | 24.574 | GC-MS |
| Gluconic acid | 333.1 | 22.268 | GC-MS |
| Glucose | 319.2 | 23.308 | GC-MS |
| Glutamic acid | 246.1 | 19.908 | GC-MS |
| Glutamine | 245.1 | 21.649 | GC-MS |
| Glyceric acid | 292.2 | 16.294 | GC-MS |
| Glycine | 276.1 | 15.923 | GC-MS |
| Glyoxylic acid | 160.0 | 10.722 | GC-MS |
| Gulonic acid | 292.2 | 24.092 | GC-MS |
| Heptanoic acid | 144.1 | 13.905 | GC-MS |
| Hexadecanoic acid | 313.3 | 24.216 | GC-MS |
| Hydrated Formaldehyde | 66.0 | 8.552 | GC-MS |
| Idose | 205.1 | 23.440 | GC-MS |
| Inositol | 305.2 | 24.958 | GC-MS |
| IS | 319.2 | 21.249 | GC-MS |
| Isoleucine | 158.1 | 15.746 | GC-MS |
| Lactic acid | 220.1 | 13.430 | GC-MS |
| Leucine | 158.1 | 15.438 | GC-MS |
| Linoleic acid | 337.3 | 25.733 | GC-MS |
| Malic acid | 233.1 | 18.356 | GC-MS |
| Mannitol | 364.2 | 22.936 | GC-MS |
| Mannose-6-Phosphate | 387.2 | 27.049 | GC-MS |
| Melezitose | 305.2 | 30.684 | GC-MS |
| Melibiose | 531.2 | 33.431 | GC-MS |
| Monomethylphosphate | 241.1 | 14.072 | GC-MS |
| myo-Inositol | 433.3 | 33.723 | GC-MS |
| myo-Inositol-1-phosphate | 315.1 | 27.834 | GC-MS |
| N-Acetylglutamic acid | 186.1 | 18.815 | GC-MS |
| Nicotinic acid | 106.0 | 15.696 | GC-MS |
| N-Methoxy-N-Amine | 160.1 | 9.531 | GC-MS |
| Norvaline | 113.1 | 9.970 | GC-MS |
| Octadecanoic acid | 341.3 | 25.991 | GC-MS |
| Octadecatrienoic acid | 235.1 | 25.783 | GC-MS |
| Octamethyltrisiloxane | 221.1 | 8.296 | GC-MS |
| Pentanoic Acid | 258.1 | 13.126 | GC-MS |
| Phenylalanine | 218.1 | 19.915 | GC-MS |
| Phloroglucinol | 327.1 | 20.173 | GC-MS |
| Phosphoric acid | 314.1 | 15.506 | GC-MS |
| Putrescine | 174.1 | 17.948 | GC-MS |
| Pyroglutamic acid | 156.1 | 18.748 | GC-MS |
| Quinic acid | 345.2 | 22.699 | GC-MS |
| Raffinose | 437.3 | 29.660 | GC-MS |
| Serine | 132.1 | 15.214 | GC-MS |
| Shikimic acid | 204.1 | 22.041 | GC-MS |
| Succinic acid | 247.1 | 15.973 | GC-MS |
| Sucrose | 450.3 | 30.001 | GC-MS |
| Tetradecane | 112.1 | 17.056 | GC-MS |
| Threonic acid | 292.2 | 19.315 | GC-MS |
| Threonine | 218.1 | 17.064 | GC-MS |
| Trehalose | 452.2 | 29.809 | GC-MS |
| Turanose | 204.1 | 29.076 | GC-MS |
| Tyrosine | 400.2 | 23.359 | GC-MS |
| Uracil | 241.1 | 16.364 | GC-MS |
| Valine | 144.1 | 14.607 | GC-MS |
| Xylobiose | 361.2 | 30.257 | GC-MS |
| Xylose | 307.2 | 20.549 | GC-MS |
| Xylulose | 307.2 | 20.724 | GC-MS |
| PG(34:2) | 745.5 | 11.235 | negative |
| PA(34:3) | 669.4 | 10.738 | negative |
| PA(36:4) | 695.5 | 10.730 | negative |
| PA(36:5) | 693.4 | 10.156 | negative |
| PA(34:2) | 671.5 | 11.260 | negative |
| PA(36:3) | 697.5 | 11.269 | negative |
| PA(34:1) | 673.5 | 11.800 | negative |
| PG(32:0) | 721.5 | 11.439 | negative |
| PA(36:6) | 691.4 | 9.576 | negative |
| PG(38:5) | 795.5 | 10.600 | negative |
| PA(43:2) | 797.6 | 11.906 | negative |
| PG(35:2) | 759.5 | 11.485 | negative |
| PG(38:6) | 793.5 | 10.049 | negative |
| PA(30:0) | 619.4 | 11.700 | negative |
| PG(36:2) | 773.5 | 11.706 | negative |
| PA(35:3) | 683.5 | 11.112 | negative |
| PI(36:2) | 861.5 | 11.328 | negative |
| SQDG(32:2) | 789.5 | 10.134 | negative |
| SQDG(38:4) | 869.5 | 10.947 | negative |
| SQDG(37:4) | 855.5 | 10.695 | negative |
| SQDG(38:6) | 865.5 | 9.939 | negative |
| SQDG(37:3) | 857.5 | 11.120 | negative |
| Hexadecyl Acetyl Glycerol | 359.3 | 10.093 | positive |
| PC(40:1) | 844.7 | 11.289 | positive |
| LysoPC(38:2) | 800.6 | 11.521 | positive |
| LysoPC(36:2) | 772.6 | 10.189 | positive |
| LysoPC(37:2) | 786.6 | 10.824 | positive |
| PC(34:2) | 758.6 | 9.585 | positive |
| PC(39:1) | 830.7 | 10.638 | positive |

## Supplementary Table 2 The ion mode, m/z, characteristic fragment ions were referenced to lipids identification.

| Ion mode | m/z | Characteristic fragment ion | Lipids |
| --- | --- | --- | --- |
| [M+H]+ | 184.07 | [C_5_H_15_NO_4_P]^+^ | PC, LysoPC |
| [M-H]- | 152.99 | [C_3_H_6_PO_5_]^-^ | PA, PG |
| [M-H]- | 171.01 | C_3_H_8_O_6_P | PG |
| [M-H]- | 204.01 | [C_6_H_10_PO_8_]^-^ | PI |
| [M-H]- | 225.01 | [C_6_H9O_7_S]^-^ | SQDG |
